# Differences in patient outcomes and chronic care management of oral anticoagulant therapy: an explorative study

**DOI:** 10.1186/1472-6963-11-18

**Published:** 2011-01-27

**Authors:** Hanneke W Drewes, Mattijs S Lambooij, Caroline A Baan, Bert R Meijboom, Wilco C Graafmans, Gert P Westert

**Affiliations:** 1 TRANZO, Tilburg University, Tilburg, The Netherlands; 2 Centre for Prevention and Health Services Research, National Institute for Public Health and the Environment, Bilthoven, The Netherlands; 3 Scientific Institute for Quality of Healthcare (IQ healthcare), Radboud University Nijmegen Medical Centre, Nijmegen, The Netherlands

## Abstract

**Background:**

The oral anticoagulant therapy - provided to prevent thrombosis - is known to be associated with substantial avoidable hospitalization. Improving the quality of the oral anticoagulant therapy could avoid drug related hospitalizations. Therefore, this study compared the patient outcomes between Dutch anticoagulant clinic (AC) regions taking the variation in chronic care management into account in order to explore whether chronic care management elements could improve the quality of oral anticoagulant therapy.

**Methods:**

Two data sources were combined. The first source was a questionnaire that was send to all ACs in the Netherlands in 2008 (response = 100%) to identify the application of chronic care management elements in the AC regions. The Chronic Care Model of Wagner was used to make the concept of chronic care management operational. The second source was the report of the Dutch National Network of ACs which contains patient outcomes of the ACs.

**Results:**

Patient outcomes achieved by the ACs were good, yet differences existed; for instance the percentage of patients in the appropriate therapeutic ranges varied from 67 to 87% between AC regions. Moreover, differences existed in the use of chronic care management elements of the chronic care model, for example 12% of the ACs had multidisciplinary meetings and 58% of the ACs had formal agreements with at least one hospital within their region. Patient outcomes were significantly associated with patient orientation and the number of specialized nurses versus doctors (p-values < 0.05). Furthermore, the overall extent to which chronic care management elements were applied was positively associated with patient outcomes (p-values < 0.05).

**Conclusions:**

Substantial differences in the patient outcomes as well as chronic care management of oral anticoagulant therapy existed. Since our results showed a positive association between overall application of chronic care management and patient outcomes, additional research is needed to fully understand the working mechanism of chronic care management.

## Background

Oral anticoagulant therapy (OAT) is one of the major causes of drug related avoidable hospitalizations [1,2]. Oral anticoagulants - chronically used to prevent thrombosis - have a narrow therapeutic range to balance the risk of haemorrhage and thrombosis [3]. Moreover, this balance is easily influenced by various factors, such as co-medication or dosage modification [4,5]. As a consequence, many health care professionals influence the effect of OAT for instance when subscribing co-medication [6]. Therefore, the management of OAT is paramount to prevent adverse events (e.g. haemorrhage or thrombosis) [7].

A broad line of research studying the initiatives to improve the management of chronic care has evolved in the last decade. Terms used for these initiatives are case management, shared care, and integrated care, but perhaps best known from an international perspective are the Chronic Care Model (CCM) and disease management, both of which were introduced first in the U.S. [7,8]. These models are implemented to improve chronic care management to realize patient-centered care in which problems like fragmentation, guideline inadherence and restricted self-management are limited.

With respect to the OAT, differences in chronic care management exist between countries. Chronic care management is provided by routine medical care (for instance in France and US) or specialized anticoagulant clinics (ACs) (for instance in Italy and the Netherlands) [9,10]. In particular, in the Netherlands, intensive follow-up of patients using oral anticoagulants is provided by 61 specialized anticoagulant clinics (ACs) at the time of our study [11]. After subscription of the oral anticoagulants by the primary physician or specialist, all patients are followed by the ACs at least every 6 weeks. The AC's specialized nurse and/or specialized physician advice on medication doses based on their gathered monitoring results. So far, previous studies have shown that ACs achieve better patient outcomes than routine medical care [3,9,12]. However, influences of differences in chronic care management between ACs regions on patient outcomes of OAT are unclear.

Since chronic care management improves patient outcomes for certain diseases [8,13,14], this study will compare the patient outcomes between AC regions taking the variation in chronic care management into account. First, we will describe differences in patient outcomes between the AC regions for patients chronically receiving OAT. Second, we will describe the differences in chronic care management between these AC regions. Finally, we will study the association between the variation in patient outcomes and the differences in chronic care management in order to reveal suggestions for quality improvement.

## Methods

Data used in this study derived from two sources which were combined. The first source is a questionnaire that was send to all ACs in the Netherlands in 2008 (response = 100%). The second source is the publicly available year report of the Dutch National Network of ACs (FNT). From these reports, the patient outcomes - which are the results of two cross-sectional measurements assessed by all ACs at two identical moments in the year - were derived.

Two relatively new ACs were excluded for this study, because their patients only include self-management patients, making comparison incorrect. The remaining 59 ACs in the Netherlands take care of more than 375.000 OAT patients users in 2008 [15], including about 99% extramural thrombosis care in the Netherlands.

### Patient outcomes

The International Normalized Ratio (INR) is used as intermediate outcome for the quality of care. The INR - a standardized transformation of the prothrombin time to assess the degree of anticoagulation [3]- is used by the ACs to determine the needed dosage of oral anticoagulants to correct the prothrombin time. The optimal target range of the INR for patients depends on the type of indication [3] and is differentiated to two indication groups by the Dutch National Network of ACs, i.e. low intensity group with INR 2.5-3.5 and high intensity group with INR 3.0-4.0. We used three different measures of the INR as patient outcome. The first indicator is the percentage of patients within the target range of the INR. Recently, the therapeutic range (a broader range including 0.5 points below the target ranges) is used besides the target range, since it is assumed to be a better measure for the quality of care considering the OAT [15]. Therefore, the percentage of long-term patients within the therapeutic range is used as second outcome variable. Additionally, the percentage of patients below the therapeutic range will be used as a quality indicator. This outcome is selected because it was recently shown that the relative risk of adverse events below an INR of 2 is higher than an INR of 3 to 5 [16,17]. In addition, we attempted to use the number of severe complications which is restricted to severe haemorrhages as quality indicator. However, we excluded this outcome measure since the reliability of the reported results considering this measure are questioned by the Dutch National Network of ACs and the authors of this paper [15].

### Chronic care management

The Chronic Care Model (CCM) of Wagner is used to identify elements of chronic care management. The CCM captures six components which all conceive elements on practice level to structure chronic care such as software applications for decision support or education for self management support [7]. The CCM elements that were included in our questionnaire were selected and made measurable based on the literature and the expert opinion of about twenty professionals working in the thrombosis field. The characteristics of the chronic care management identified with the questionnaire are described by five components of the CCM namely: health care organization (i.e. the organizations' focus on chronic care for instance by incident reporting system); self-management support (i.e. supporting patients to manage their condition for instance by self-management education); delivery system design (i.e. the organization of providing care such as other roles/teams); decision support (i.e. integration of evidence based clinical guidelines into practice for example by a reminder system) and clinical information system (i.e. systems that support the information exchange) (table 1). The identified chronic care management elements applied by the AC regions are hypothesized to improve the chronic care management and as a consequence the patient outcomes as suggested by the CCM. Although the sixth component of the CCM - community resources and policies - is relevant for chronic care management (e.g. legislation to allow self management), this is not taken into account in this study because the variation in this element differs on another level than the level of analysis of this study, i.e. the AC regions.

**Table 1:** Differences between AC regions in chronic care management categorized by the CCM elements (N = 59)

	N	mean (sd)
**Health care organization**		
Client board	56	
Quality manager	58	
Quality improvement system (none/IKA or VIM/both)	1/10/48	
Insight in telephonic waiting times(proxy patient orientation)	24	
Accreditation (no/once/more than once)	14/13/32	
		
**Self management support**		
Percentage of self management patients		5.0 (4.9)
Self-management support with webbased application	15	
Frequency of self management control by AC		3.8 (1.4)
		
**Delivery system (re)design**		
Regional multidisciplinary meeting	7	
Specialized nurses versus doctors		8.7(11.3)
		
**Decision support**		
Formal agreements with hospitals	34	
Software used for dosage advice	57	
Info to AC about prescribed interacting drugs (no. always and mean (scale never-always: 0-3))		
Info from the pharmacist about starting	28	2.4 (0.6)
Info from the dispensing GP about starting	11	1.5 (1.0)
Info from the pharmacist about change/discontinue	12	1.6 (1.0)
Info from the dispensing GP about change/discontinue	4	1.0 (1.0)
Information from the hospital (scale slow-fast: 0-10)		1.1 (1.5)
		
**Clinical information systems**		
Communication medium (telephone or other with INR > 8)	44	
Webbased patient information for dosage advice	11	

GP (general practitioner), IKA (system to report ideas, complaints and deviations), INR (International normalized ratio), VIM (safe reporting of incidents).

### Data analysis

A combination of descriptive analysis and regression analysis was used to perform this study. The variation in patient outcomes and the elements of chronic care management were studied with descriptive analyses. Subsequently, data were subjected to regression analysis to identify to what extent the differences in patient outcomes are associated with chronic care management activities. The regression analysis were controlled for the type of reagent (the use of Innovin versus other reagent) since previous research showed that use of Innovin may systematically give different results on the measured INR [18]. The associations between the use of CCM and quality of care are studied in two different ways.

First, we studied the use of each separate element of the CCM and its association with quality of care. Items of CCM were excluded from the regression analysis if the variation was too small (i.e. less than 10%).

Second, we constructed a scale representing the use of all elements of the CCM. Since Wagner et al. argue that the use of more elements of CCM is associated with a stronger improvement of the quality of care, this may be a more appropriate way to study the associations of interest [19]. The CCM variables were transformed into a z-distribution to aggregate the items to the related components of the CCM by computing the mean. Subsequently, we determined the relative use of the components of the CCM. The components of Wagner were included as a score in the construct when its use was more than 0 on the z-scale, indicating that it was used more than average by the AC region. The ensuing scale represented the number of CCM components which are used more by a thrombosis clinic than by other thrombosis clinics. It can be interpreted as a count of the relative use of the CCM components: the higher the score, the more CCM components are used.

## Results

### Quality of care

**Figure 1:**
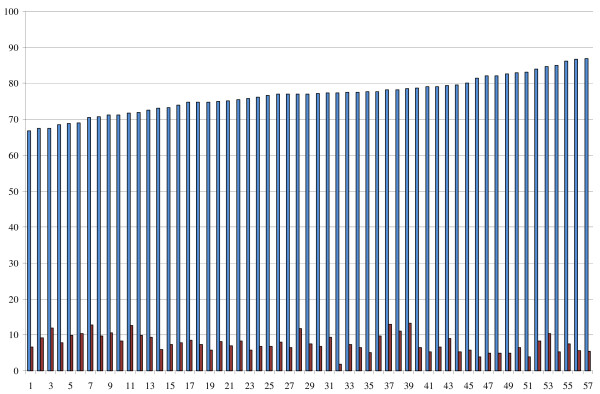
**Patient outcomes on the therapeutic range (N = 57)**. Blue bar shows the percentage of patients within the appropriate therapeutic range per AC region. Red bar shows the percentage of patients below the appropriate therapeutic range per AC region.

Results showed that the percentages of patients within and below the therapeutic range varied. Figure 1 visualizes these patient outcomes for each AC region. The number of patients within the appropriate therapeutic range varied between AC regions from 66.7% to 86.8% (mean = 76.8; sd = 5.1). Furthermore, the number of patients below the appropriate therapeutic range varied from 1.8% to 13.4% (mean = 7.8; sd: 2.6) of the patients between the AC regions. The number of patients within the appropriate target range varied from 48.5% to 70.7% (mean = 59.1; sd = 5.8). This implies that the chance to be in the correct target or therapeutic range can be 20% higher in one AC region compared to another.

### Chronic care management

The differences in chronic care management between the AC regions, categorized to components of the CCM, are shown in table 1. All ACs reported to have a quality manager and almost all a client board which is mandatory in the Netherlands. Differences are shown in the component 'health care organization' considering quality improvement system and patient orientation. In addition, more than half of the ACs have been accredited more than once which implies consistency of their quality of care.

The component 'delivery system design' showed that 7 of the 59 thrombosis clinics reported to participate in regional multidisciplinary meetings in 2007. The ratio of specialized nurses versus doctors - measured in full-time equivalent - showed a mean ratio of 8.7. This implies that the average number of full-time equivalents (FTE's) of the specialized nurses is 8.7 times higher than the number of FTE's of the doctors. Especially the number of specialized nurses differed; ranging from 0 specialized nurses (which is the case for 9 ACs) to 15 specialized nurses working at the AC.

Furthermore, the component 'decision support' was applied in most AC regions (n = 57) by using a software system which propose dosage advices that could give the doctors a direction. Formal agreements between the hospital and the ACs are less frequently reported; 34 of the ACs had formal agreements with at least one hospital of their region.

In addition, the characteristics of the component 'clinical information system' showed that 44 of the ACs always contact the physician about a INR above 8.0 (which is advised by the Dutch National Network of ACs), 11 of the ACs use a web based dosage system, less than half of the ACs are always informed about interacting drugs by the pharmacist.

### The association between quality of care and CCM

First, the associations between the individual chronic care elements and the quality of care were studied (table 2). These regression analyses were corrected for the type of reagent (Innovin or other reagent) which was significantly associated with quality of care (p = 0.008) and is likely to distort the associations we are interested in. The analysis showed that the variables 'insight in waiting times' (as proxy for patient orientation) and the ratio 'specialized nurses versus doctors' were associated with a significant higher number of patients within the therapeutic range. Furthermore, only the ratio 'specialized nurses versus doctors' is statistically significant associated with the number of patients within the target range. No statistically significant associations were observed with the number of patients below the therapeutic range.

**Table 2:** Association between patient outcomes and chronic care management elements categorized by the CCM elements (N = 57)

	Therapeutic range		Target range	
	B	p-value	B	p-value
**Health care organization**				
Client board	-3.001	0.304	-3.096	0.359
Quality manager	NA	NA	NA	NA
Quality improvement system (none/IKA or VIM/both)	0.901	0.550	1.008	0.562
Insight in telephonic waiting times (proxy patient orientation)	2.524	0.050*	1.883	0.210
Accreditation (no/once/more than once)	1.225	0.124	1.265	0.170
				
**Self management support**				
Percentage of self management patients	21.450	0.109	8.097	0.604
Self-management support with webbased application	-2.583	0.077	-3.103	0.065
Frequency of self management control by AC	0.616	0.198	0.259	0.641
				
**Delivery system (re)design**				
Regional multidisciplinary meeting	-0.255	0.898	-2.135	0.352
Specialized nurses versus doctors	0.131	0.035*	0.166	0.020*
				
**Decision support**				
Formal agreements with hospitals	0.097	0.941	0.350	0.817
Software used for dosage advice	NA	NA	NA	NA
Info to AC about prescribed interacting drugs (no. always and mean (scale never-always: 0-3))				
Info from the pharmacist about starting	0.337	0.774	1.396	0.301
Info from the dispensing GP about starting	0.127	0.848	0.921	0.225
Info from the pharmacist about change/discontinue	-0.135	0.841	-0.923	0.231
Info from the dispensing GP about change/discontinue	-0.276	0.696	0.004	0.996
Information from the hospital (scale slow-fast: 0-10)	-0.090	0.893	0.463	0.364
				
**Clinical information systems**				
Communication medium (telephone or other with INR > 8)	2.437	0.099	2.136	0.213
Webbased patient information for dosage advice	0.346	0.844	-1.303	0.521

GP (general practitioner), IKA (system to report ideas, complaints and deviations), INR (International normalized ratio), NA (not assessed), VIM (safe reporting of incidents).

Second, the association between the overall use of chronic care management and the patient outcomes was studied. The analysis showed that the use of more CCM components was positively associated with better patient outcomes since the number of components was associated with a higher percentage of patients within the therapeutic range (B = 1.248; p = 0.017) and target range (B = 1.358; p = 0.024). The use of more CCM components was not significantly associated with the percentage of patients below the therapeutic range (B = -0.485; p = 0.073).

## Discussion

This study was performed to identify the differences in patient outcomes and their relationship with chronic care management of OAT. Although the Netherlands manage OAT with specialized clinics as is recommended by the ACCP guidelines and achieve a good quality of care according to the criteria of the Dutch National Network of ACs, remarkable differences exist. The percentage of patients in the correct INR ranges differed with more than 20%-point. Furthermore, differences existed in the application of chronic care management which was measured with elements based on the components of the chronic care model (i.e. the health care organization, self-management support, delivery system design, decision support and clinical information system). Two chronic care management elements, i.e. patient orientation and the ratio of specialized nurses versus doctors, were associated with some patient outcomes. Moreover, the overall use of chronic care management elements is positively associated with the patient outcomes.

To our knowledge, no other comparative study of the differences on the quality of OAT between regions was performed on a national level before. It was already shown in previous research that the quality of OAT provided by ACs is higher than OAT provided by routine medical care. For example, Ansell and colleagues showed that the percentage of patients within the correct INR ranges and time-in-range is higher in anticoagulant clinics in Spain and Italy than in routine medical care in France, U.S. and Canada [9]. Our study showed that differences in quality of OAT exist between ACs which suggests that even the ACs that already provide a relatively high quality of care compared with routine medical care [9], could improve their quality of care. Furthermore, our results showed differences in the percentage patients below the appropriate therapeutic range (range 1.8% to 13.4%) which should be further studied as the relative risk for adverse events was suggested to be frequently underestimated [16,17].

The identified differences in chronic care management offered an opportunity to explore whether these could be associated with the quality of OAT. Only two chronic care management elements, i.e. patient orientation and the ratio of specialized nurses versus doctors, were significantly associated with patient outcomes while the other elements were not. It could be hypothesized that patient orientation and ratio of specialized nurses versus doctors results in more patient centered care since there is more time spent per patient and therefore more likely to deliver good care. However, the data of this study were not specific enough to test the hypothesis and should be further studied.

Furthermore, we found an association between the use of more CCM components and better patient outcomes. This is in line with results of earlier published meta-analyses of clinical trials [8,13,14], that showed an association between the differences in patient outcomes and chronic care management. Moreover, our results seem to confirm that only when more components of the CCM are used, this will result in better care, while fragmentary use of the CCM is unlikely to improve care.

As a consequence, additional insight in the use and validation of a construct variable for the overall application of chronic care management can be useful since the effect is assumed to be achieved by the combination of initiatives instead of certain elements. Some instruments were developed to assess the overall extent of chronic care management, however, the validation of these instrument are limited and not performed for OAT [20,21]. A validated instrument to measure the construct variable would be useful for the professionals to gather insight in the needs for quality improvement regarding chronic care management.

Meanwhile, the complexity of the chronic care management should be taken into account. Chronic care management is a social construct which effectiveness is influenced by more factors than the number of components such as the implementation and the integration of the chronic care model components [22-24]. As chronic care management seemed to be associated with the patient outcomes for OAT, more insight in the working mechanism of chronic care management is needed for quality improvement. In particular, qualitative studies are required to explore the association between chronic care management and patient outcomes in more detail. First, as chronic care management is complex and underlying mechanisms are not fully understood, additional qualitative research should be performed to identify the true needs for quality improvement [23,25]. Second, oral anticoagulants is not a static field, but is evolving over time. For instance, two recently developed ACs only include relatively healthy patients who are performing self-management. For comparability these ACs were not included in our analysis. Although these two ACs provide care to only a relatively small number of patients, this new organizations could inspire the traditional ACs.

Findings of this study must be interpreted in the light of several limitations. First, the questionnaire was sent out by the Health Care Inspectorate (IGZ) which could cause bias especially social desirability bias which might have resulted in an underestimation of the association between chronic care management and patient outcomes. However, the respondents were aware that this questionnaire aimed to explore the chronic care management of OAT on regional level instead of focusing on the ACs alone. Next, the highly developed documentation of the ACs on national level is in contrast with the scarcely developed documentation in the clinical setting. As a consequence, gaps exist in the follow-up of patients (e.g. INR values around hospitalization). These gaps could not be analysed and controlled for. Thus they may have affected the association between patient outcomes and elements of the chronic care model under study. Furthermore, chronic care management could not be measured by a validated instrument since these are not yet available for OAT. However, we selected and made the elements measurable based on the literature and the expert opinions of about twenty professionals working in the thrombosis field. Finally, the analyses of this study were limited to quality measures reported on national level. This implies that analyses were performed on the organization level without case mix corrections due to a lack of information about patient characteristics such as age and co-morbidity which are not systematically registered by the ACs. We only could correct for the type of reagent, other variations in data gathering for the reported outcome measures could not be eliminated. Yet, the used data-registration of the Dutch National Network of ACs on national level is unique worldwide and gives opportunities to gather more insight in the needs for quality improvement. Therefore, AC regions should be stimulated to gain more insight in the delivery of chronic care management and their influences on the patient outcomes.

## Conclusions

Although, the Netherlands manage oral anticoagulation therapy (OAT) as is recommended by Ansell and colleagues in the ACCP guidelines - i.e. systematic and coordinated OAT incorporating patient education, systematic INR testing, tracking, follow-up and good patient communication, results and dosing decisions by specialized clinics - we observed differences in quality of care and chronic care management which implies opportunities to improve the OAT care management. The patient outcomes seemed to be associated with the overall application of chronic care management. To get more insight in the needs of quality improvement, additional data about the working mechanism of chronic care management with respect to patient outcomes are required.

## Competing interests

The authors declare that they have no competing interests.

## Authors' contributions

All authors contributed to the conception, design, interpretation of data, drafting and editing of the manuscript. HD and WG acquired the data; HD and ML analyzed the data. All authors have read and approved the manuscript.

## Pre-publication history

The pre-publication history for this paper can be accessed here:

http://www.biomedcentral.com/1472-6963/11/18/prepub
